# Draft genome sequences of a putative novel subspecies of *Streptococcus* isolated from wastewater

**DOI:** 10.1128/mra.01179-25

**Published:** 2026-04-14

**Authors:** Lei Wang, Xingchao Zhao, Liyu Wu, Quan Wen, Zhaohai Liang, Yong Zhang, Wan Liu

**Affiliations:** 1 Microbiology Laboratory, Shenzhen Center for Disease Control and Prevention 568734, Shenzhen, Guangdong Province, China; 2 Microbiology Laboratory, Futian District Center for Disease Control and Prevention, Shenzhen, Guangdong Province, China; 3 Physical and Chemical Testing Laboratory, Futian District Center for Disease Control and Prevention, Shenzhen, Guangdong Province, China; Nanchang University, Nanchang, Jiangxi, China

**Keywords:** *Streptococcus*, wastewater, genome, novel subspecies

## Abstract

We report two *Streptococcus* strains (FT1-55/FT1-106) isolated from Shenzhen wastewater. Their genomes (~2.35 Mb; GC ~38.5%) show high relatedness (average nucleotide identity: 97.8%–98.0%) to *Streptococcus orisratti* yet form a distinct phylogenetic clade, supporting their proposal as a novel subspecies, *Streptococcus orisratti* subsp. *futianensis* subsp. nov.

## ANNOUNCEMENT

The genus *Streptococcus* comprises a diverse group of gram-positive bacteria within the order *Lactobacillales* and the family *Streptococcaceae*. As of the latest update on 25 July 2025 (https://lpsn.dsmz.de/genus/streptococcus), the genus *Streptococcus* includes 142 validly published species (1, 2). *Streptococci* cause infections ranging from pneumonia and meningitis to skin and urinary tract diseases (3). Wastewater systems offer critical insights for expanding reference databases and tracking emerging resistance determinants (4). Here, we report the genome sequences of two *Streptococcus* sp. isolates obtained from wastewater in Shenzhen, China. Based on genomic and phylogenetic analyses, we describe a putative novel subspecies within the genus *Streptococcus*.

Wastewater samples were collected from the inflow of sewage treatment plants in Futian District of Shenzhen between July 2023 and June 2024 (*n *= 100). After centrifugation of 250 mL wastewater aliquots individually, pellets were resuspended, subjected to a 100-fold dilution, and spread-plated (100 µL) on Brain Heart Infusion agar and Reasoner’s 2A agar (HiMedia), then incubated aerobically at 25–40℃ for 3–7 days (5). Isolated colonies were then obtained through quadrant streaking. Two pure cultures, designated *Streptococcus* sp. FT1-55 and *Streptococcus* sp. FT1-106 (hereafter FT1-55 and FT1-106), were isolated from two independent samples collected on 4 and 18 July 2023.

For whole-genome sequencing, genomic DNA was extracted using previously described methods (3). Library preparation was performed using the NEBNext DNA library prep mastermix kit (NEB, Inc.), and paired-end sequencing (2 × 150 bp) was conducted on an Illumina NovaSeq 6000 platform (Novogene Bioinformatics Technology, Beijing, China). Quality assessment of 9,757,872 (FT1-55) and 9,797,882 (FT1-106) raw reads was performed with FastQC v0.11.9 (6), followed by trimming and filtering using Trimmomatic version 0.39 (7). High-quality reads were assembled *de novo* with SPAdes v3.14.1 (8). Genome annotation was carried out using the NCBI Prokaryotic Genome Annotation Pipeline version 6.9 (9). All tools were run with default parameters. Assembly statistics and annotation features were detailed in Table 1.

**TABLE 1:** Summary of assembly and annotation features of two *Streptococcus* sp. isolates from wastewater

	*Streptococcus* sp. FT1-55	*Streptococcus* sp. FT1-106
BioProject ID	PRJNA1236501	PRJNA1236501
BioSample ID	SAMN47389735	SAMN47390754
NCBI RefSeq Assembly	GCF_049314995.1	GCF_049314965.1
Total no. of reads	1,671,468	1,671,687
Genome size (bp)	2,367,077	2,333,986
No. of contigs	137	167
G + C content (%)	38.54	38.51
N50(bp)	62,701	55,055
L50	12	16
Genome Coverage (×)	216	219
Genes (total)	2,438	2,410
CDSs (total)	2,378	2,352
No. of tRNAs	53	51
No. of rRNAs	3	4
CRISPR Arrays	1	1

**Fig 1:**
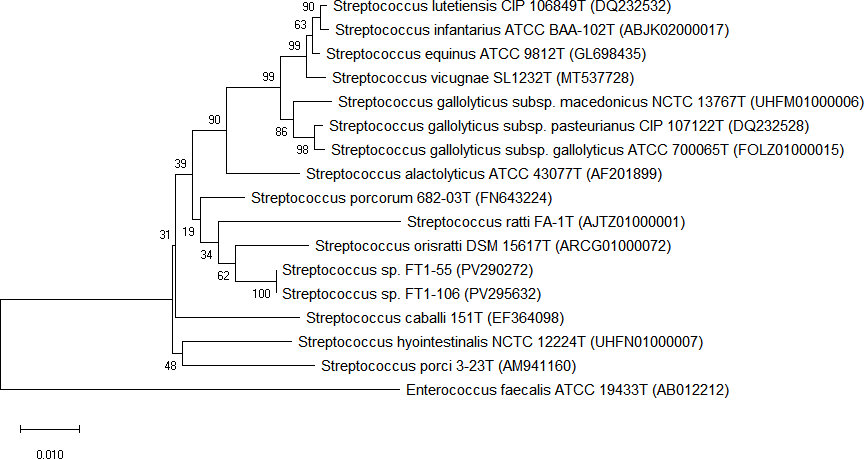
Neighbor-joining tree based on the 16S rRNA gene sequences. The tree shows the taxonomic position of strains FT1-55, FT-106, and other closely related species. Bootstrap values (>50%) from 1,000 replicates are shown. *Enterococcus faecalis* ATCC 19433^T^ was served as the outgroup. Scale bar indicates 0.01 substitutions per nucleotide position.

Additionally, digital DNA–DNA hybridization (dDDH) was calculated using the Genome-to-Genome Distance Calculator (GGDC v.3.0) with the recommended formula 2 (10). The average nucleotide identity (ANI) value was estimated using Orthologous ANI (OrthoANI) (11). The EzBioCloud 16S database v.2025.04.21 (https://www.ezbiocloud.net/) was used for sequence comparisons (12). The phylogenetic tree (Neighbor-Joining, NJ) based on 16S rRNA gene sequences was constructed with MEGA version 11 software (http://www.megasoftware.net) (13). Complete 16S rRNA genes were extracted from assembled genomes. Comparative genomic analysis of strains FT1-55 and FT1-106 against their closest relatives revealed 16S rRNA gene sequence similarity below 98.7% and dDDH values between 22.8% and 85.2%, supporting their preliminary classification as distinct variants within the group (3). Notably, both strains exhibited high genomic relatedness to *Streptococcus orisratti* DSM 15617ᵀ (ANI: 97.77% and 97.95%), despite showing lower ANI values (73.35%–78.14%) against other closely related strains. Furthermore, they formed a distinct clade in the 16S rRNA gene-based phylogenetic tree (Fig. 1). Based on this polyphasic evidence, we propose that these strains represent a novel subspecies, for which the name *Streptococcus orisratti* subsp. *futianensis* subsp. nov. is proposed.

## Data Availability

The genome sequence of *Streptococcus* sp. FT1-55 and FT1-106 are available under the following: BioProject accession number PRJNA1236501, BioSample accession numbers SAMN47389735 (for FT1-55) and SAMN47390754 (for FT1-106), GenBank assembly accession numbers GCF_049314995.1 (for FT1-55) and GCF_049314965.1 (for FT1-106), 16S rRNA gene accession numbers PV290272 (for FT1-55) and PV295632 (for FT1-106). Raw sequencing data is accessible in the Sequence Read Archive (SRA) under accession numbers SRR35213496 (for FT1-55) and SRR35213495 (for FT1-106).
